# Assessment of vitamin K levels in women with intrahepatic cholestasis of pregnancy

**DOI:** 10.1186/s12884-022-04875-w

**Published:** 2022-07-01

**Authors:** Maria Cemortan, Irina Sagaidac, Olga Cernetchi

**Affiliations:** Department of Obstetrics and Gynecology, Nicolae Testemitanu State University of Medicine and Pharmacy, Chisinau, Republic of Moldova

**Keywords:** Intrahepatic cholestasis of pregnancy, Obstetric cholestasis, ICP, Pregnancy

## Abstract

Intrahepatic cholestasis of pregnancy is a disorder characterized by pruritus and elevated liver function tests and bile acids. Poor vitamin absorption and, as a result, hypovitaminosis K can occur as a result of the pathology. Given the known effects of vitamin K, the authors considered that hypovitaminosis K could increase the risk of coagulopathic hemorrhage in pregnant women. The study revealed that 59.2% of women with intrahepatic cholestasis of pregnancy were diagnosed with hypovitaminosis K; however, 98.6% of women had normal coagulogram indices. Thus, coagulogram markers are more likely to indicate vitamin K activity than its actual level.

## Background

Intrahepatic cholestasis of pregnancy (ICP) is a liver disease with a global incidence of 0.5–1% [1]. The onset of cholestasis gravidarum is characterized by the appearance of skin pruritis that cannot be explained by other causes [2]. A crucial diagnostic criterion in ICP is the assessment of serum bile acid (BA) levels and liver function tests. Assessment of serum BA levels is considered the definitive biochemical marker in the diagnosis of ICP and monitoring of ICP patients’ condition [2]. Based on BA values, cholestasis gravidarum can be classified as mild (BA 10—39 μmol/L) and severe (BA ≥ 40 μmol/L) [3].

Vitamin K is a group of fat-soluble vitamins. There are two forms of vitamin K found in food: vitamin K1 (phylloquinone) and vitamin K2 (menaquinone). The remaining subtypes are synthetic forms [4]. Menaquinones are named based on the length of their unsaturated isoprenyl side chains, ranging from MK-4 to MK-13 [5]. MK-4, MK-7, and MK-9 are the most investigated variants of vitamin K2 [4]. Vitamin K1 is the most common type in the human body, and it can be obtained through foods like green vegetables, while egg yolks, chicken, beef, vegetables, and fermented foods are high in vitamin K2. Furthermore, vitamin K2 is produced by the gut microbiota via receptors (class B type I and Niemann-Pick C1-Like 1) that have recently been identified as regulators of vitamin K absorption in the intestine [6, 7].

In the small intestine, vitamin K is captured by bile salts, followed by absorption into enterocytes, after being incorporated by specific lipoproteins (which contain apolipoprotein-A and apolipoprotein-B48) and secreted into the lymphatic system and blood. All forms of vitamin K, particularly vitamin K1 and MK-7, then reach the liver. Chylomicrons, which contain vitamin K and lipoproteins, enter hepatocytes by endocytosis and are conjugated with apolipoprotein-B100 before returning to the bloodstream. These molecules undergo changes in blood circulation, characterized by the addition and removal of apolipoprotein particles. LDL cholesterol particles transport vitamin K molecules through the bloodstream, where they are taken up by LDL receptors in target tissues (such as the brain, heart, arteries, cartilage, and bones) [8, 9].

Vitamin K is a coenzyme for vitamin K-dependent gamma-glutamyl carboxylase, an enzyme involved in hemostasis, bone metabolism, and other processes [4]. During development, gamma-glutamyl carboxylase is expressed in the central nervous system. The authors propose that vitamin K plays a crucial function in central nervous system myelin production. In addition, vitamin K antagonists (such as Warfarin) can cause malformations of the fetus' central nervous system, resulting in mental retardation. These findings show that vitamin K may play a function in brain development through the prenatal period [10]. At the same time, vitamin K is one of the co-factors that determine the blood clotting process, acting through prothrombin (factor II), proconvertin (factor VII), Christmas factor (IX) and Stuart-Prower factor (X). Therefore, vitamin K has an essential role in the activation of blood-clotting proteins [11].

Gastrointestinal disorders such as celiac disease, cystic fibrosis, ulcerative colitis, and cholestasis, including intrahepatic cholestasis of pregnancy, can impair pancreatic and/or biliary functioning, resulting in lipid malabsorption mechanisms. These conditions can lead to poor vitamin absorption and, as a result, hypovitaminosis K [12]. Given the known effects of vitamin K, the authors hypothesized that a risk of coagulopathic hemorrhages in pregnant women could be induced by a vitamin K deficiency. Vitamin K malabsorption can be caused by steatorrhea, which is a direct but uncommon complication of ICP. Traditionally, vitamin K levels have been determined indirectly by assessing surrogate markers such as prothrombin time, although prothrombin time is more likely to suggest vitamin K activity than its actual level. As a result, prothrombin time prolongation is a late indicator of hypovitaminosis K. Nevertheless, this method may understate the exact prevalence of vitamin K deficiency [13].

## Material and methods

The prospective cohort study was carried out by assessing 71 pregnant women with intrahepatic cholestasis of pregnancy.

Pregnant women, over 22^+0^ weeks of gestation, with a confirmed diagnosis of ICP, which was established according to clinical features and laboratory test results (level of serum BA ≥ 10 μmol/L) were eligible for the study. Women with known coagulopathy, preeclampsia, HELLP-syndrome, acute hepatitis, and drug-induced liver injury were excluded from the study. In addition, we excluded women with epilepsy to rule out the influence of anti-epileptic medication on vitamin K absorption.

The women’s vitamin K levels (vitamin K1, vitamin K2 MK4, vitamin K2 MK7), coagulogram indices, and blood loss during delivery were assessed. The vitamin K levels were assessed by high performance liquid chromatography (HPLC). Assessment of total blood loss during delivery was carried out according to international guidelines, with samples collected in graduated vessels and used sterile material weighed. The dietary habits were assessed based on the food frequency questionnaire (FFQ), designed to estimate the usual eating pattern of the main food groups: cereals, fruits, vegetables, milk and dairy, meat, fish, nuts and seeds, cooking fats, sweets and fast food. In addition, the FFQ included questions about vitamin and supplements intake during pregnancy. Aside from that, the women's body mass index (BMI) before pregnancy was calculated, as well as a weight gain assessment scale during pregnancy were used to rule out the possibility of poor nutrition, which might cause low alimentary intake of vitamin K.

Ethical approval was granted by the Ethics Committee Review Board of the *Nicolae Testemitanu* State University of Medicine and Pharmacy of the Republic of Moldova. Written informed consent was obtained from all participants. All information was kept confidential.

After collection and checking for completeness, the data was coded and entered into the IBM SPSS Statistics 21 version and exported for further analysis using the functions and modules of the IBM SPSS Statistics 21 and GraphPad Software. The arithmetic means and standard deviation (M (SD)) were calculated to describe the numerical indicators. For a distribution different from the normal one, the median (Me), as well as the interquartile range (Q1; Q3) were calculated. To compare categorical variables in groups, the χ^2^ test was used with Yates' correction. A *t* test was used to compare the means of two groups (mild and severe forms of the condition). A *p*-value < 0.05 was considered statistically significant.

## Results

The age of pregnant women included in the study was 18–43 years. The average age of women was 29.5(6.3) years. Multiple pregnancy was found in 8/71 (11.3%) cases. Biliary acid levels in women were assessed in order to determine the severity of ICP. A mild condition was diagnosed in 50/71 (70.4%) cases and a severe condition in 21/71 (29.6%) cases. The possible nutritional component of vitamin intake was analyzed. Using the FFQ, participants self-assessed their diet. Thus, 81.7% of women consider their diet to be well equilibrated. However, the authors recognize that this is a subjective measurement. The average BMI for study participants before pregnancy was 24.1 (3.8), Me 23.7 (21.3;25.7). Therefore, 50/71 (70.4%) participants were in normal weight range before pregnancy, there were no cases of underweight BMI range in the study. Mean weight gain during pregnancy was 10.6 (5.9), Me 10 (7;13) kilos. All participants had normal values of weight gain during pregnancy, considering their initial BMI.

**Fig. 1:**
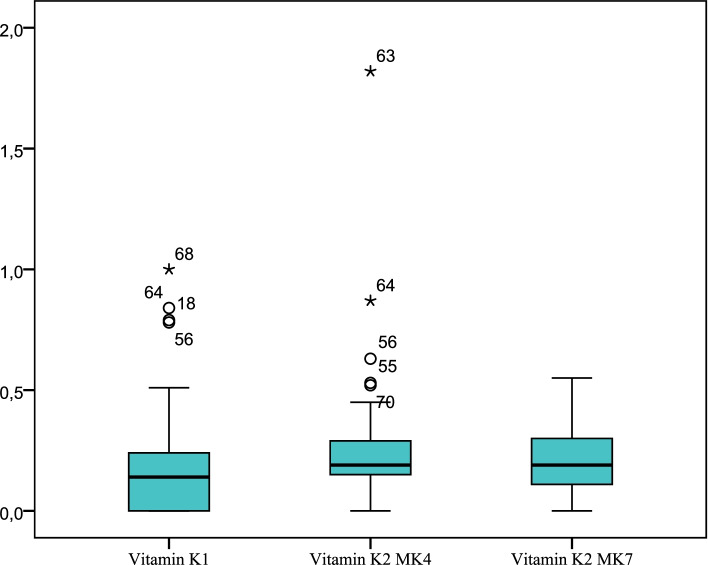
Levels of vitamin K fractions in women included in the study (µg/L)

In line with the study’s aim, vitamin K levels, including vitamin K1(reference values: 0.13–1.19 µg/L), vitamin K2 MK4 (reference values: 0.1–0.86 µg/L) and vitamin K2 MK7 (reference values: 0.1–0.82 µg/L) were assessed among participants. The mean value of studied fractions was 0.18 (0.21), Me 0.14 (0.0;0.24) µg/L for vitamin K1, 0.25 (0.23), Me 0.19 (0.15;0.30)µg/L for vitamin K2 MK4 and 0.19 (0.13), Me 0.19 (0.11;0.30) µg/L for vitamin K2 MK7 (Fig. 1).

**Fig.2:**
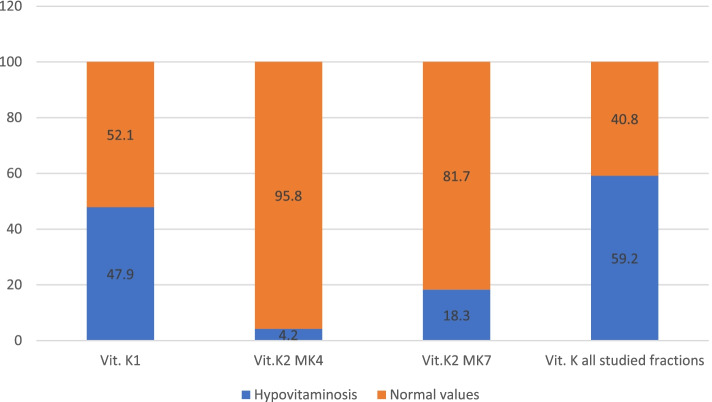
Rate of hypovitaminosis K fractions in women included in the study (%)

Despite the fact that there were normal average levels of the studied vitamin K groups, it was found that normal values of all the assessed vitamin K fractions occurred in 29/71 (40.8%) cases (Fig. 2).

**Fig. 3:**
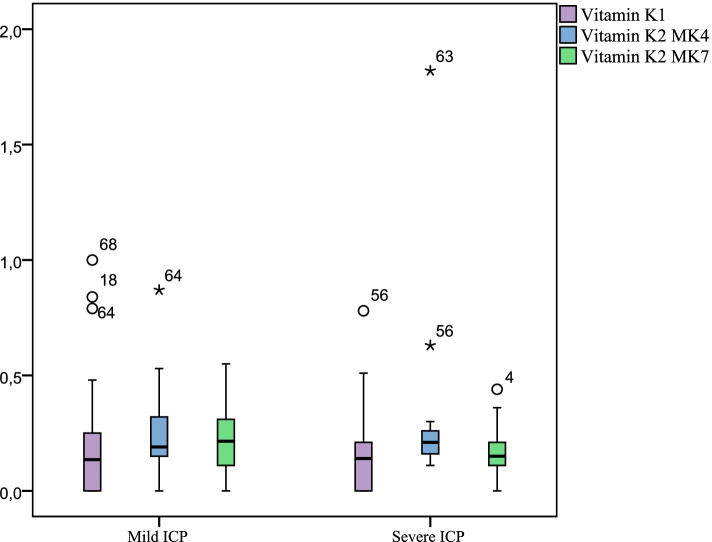
Levels of vitamin K fractions in women with mild and severe condition (µg/L)

Besides that, we were interested in how the severity of the condition influenced the vitamin K level in women, included in the study. Hence, the study revealed the mean value of vitamin K1 was 0.18 (0.22), Me 0.13 (0.0;0.25) µg/L in mild ICP and 0.17 (0.19), Me 0.14 (0.0;0.22) µg/L in severe condition. In women with mild ICP mean vitamin K2 MK4 level was0.23 (0.15), Me 0.19 (0.15;0.32) µg/L and in severe ICP it was 0.29 (0.36), Me 0.21 (0.15;0.27) µg/L. Thus, mean vitamin K2 MK7 level in mild condition was 0.21 (0.14), Me 0.21 (0.11;0.31) µg/L, mean level of vitamin K2 MK7 in severe ICP was 0.16 (0.11), Me 0.15 (0.10;0.22) µg/L, Fig. 3.

Analysis of the contingency relation between the level of biliary acids and hypovitaminosis of all studied fractions of vitamin K in women with ICP revealed no statistically significant association between hypovitaminosis K and the severity of the condition, Table 1.

**Table 1:** Contingency between level of biliary acids and hypovitaminosis of studied fractions of vitamin K in women with ICP

#	Criteria	Mild ICP *n* = 50 (abs., %)	Severe ICP *n* = 21 (abs., %)	χ^2^	p
1	Vitamin K1				
	Hypovitaminosis	25 (50.0%)	9 (42.9%)	0.084	0.7721
	Normal values	25 (50.0%)	12 (57.1%)		
2	Vitamin K2 MK 4				
	Hypovitaminosis	3 (6.0%)	0	0.251	0.6166
	Normal values	47 (94.0%)	21 (100.0%)		
3	Vitamin K2 MK 7				
	Hypovitaminosis	9 (18.0%)	4 (19.0%)	0.011	0.9170
	Normal values	41 (82.0%)	17 (81.0%)		
4	Vitamin K – all studied fractions				
	Hypovitaminosis	30 (60.0%)	12 (57.1%)	0.050	0.8231
	Normal values	20 (40.0%)	9 (42.9%)		

In order to estimate the presence and severity of coagulation disorders in women included in the study, we conducted a comparative analysis of the coagulogram indices: fibrinogen (reference values: 3.7–6.2 g/L), prothrombin by Quick (reference values: 70–120%) and international normalized ratio (INR) (reference values: 0.8–1.4). Thus, it was found that vast majority of women had normal coagulogram. However, 1/71 (1.4%) case of modifications in blood clotting was found with the following values: fibrinogen – 1.9 g/L, prothrombin by Quick – 65.6%, INR –1.53. Therefore, we compared mean level of coagulogram indices in women with mild and severe ICP, Table 2.

**Table 2:** Mean level of coagulogram indices in women with mild and severe ICP

#	Criteria	Mild ICP *n* = 50 M (SD)	Severe ICP *n* = 21 M (SD)	t	P
1	Fibrinogen, g/L	4.5 (0.9)	4.7 (1.3)	0.7453	0.4586
2	Prothrombin by Quick, %	110.1 (18.4)	119.6 (14.3)	2.1103	0.0385
3	INR	0.99 (0.11)	0.95 (0.11)	1.3984	0.1665

In order to assess possible complications in labor, the timing and method of delivery, the frequency and category of cesarean sections, and blood loss during childbirth were studied. The analysis revealed increased frequency of preterm births among women with ICP (19/71 (26.8%)), although at term births prevailed – 52/71 (73.2%) cases. Vaginal births were more common (41/71 cases (57.7%)), although the rate of cesarean sections was 42.3% (30/71) in women with ICP. Average estimated total blood loss was 480 (190) ml. At the same time, we compared estimated total blood loss in women who gave birth naturally with those who had a c-section. Therefore, average estimated blood loss in vaginal delivery was 376 (133) mL vs 688 (117) mL in women who had a cesarean section. It should be noted that in 4/41 (9.7%) cases the total blood loss during vaginal delivery was more than 500 mL, among which 1/4 (25.0%) women had massive blood loss (1000 mL), managed conservatively.

## Discussions

There is not enough evidence in the literature to characterize the effects of vitamin K during pregnancy. Vitamin K's effects on chronic renal disease, coronary heart disease, osteodystrophy, osteoporosis, and other disorders have been well researched in the general population [4, 8]. Furthermore, there is evidence of a probable involvement for hypovitaminosis K in the pathogenesis of Alzheimer's disease, although further research is required [14]. At the same time, because vitamin K is required for bone metabolism, it has been used as a safe treatment for pregnancy-related osteoporosis [15, 16]. Vitamin K2 has been demonstrated to suppress some cancer cells without causing side effects, making it a promising chemical for cancer prevention and treatment [12]. Vitamin K2 has been shown in clinical tests to have the potential to improve the prognosis of cancer patients [17].

Vitamin K deficiency can pose a significant health risk to both the mother and the fetus, which can cause bleeding, especially in newborns. Hemorrhage in these cases occurs due to low levels of prothrombin, which is a substance that depends upon vitamin K. Vitamin K deficiency may be considered clinically significant when prothrombin time increases significantly due to decreased prothrombin activity in the blood [4]. Vitamin K deficiency is extremely rare in the general adult population, although it can occur in cases of vitamin malabsorption due to an associated condition. The role of vitamin K during pregnancy is mostly unknown. However, nutritional requirements generally increase during pregnancy, so the risks of clinically significant nutritional deficiencies also increase, especially among women with poor nutrition [18]. Moreover, maternal malnutrition not only has short-term effects but also causes various fetal pathologies that can manifest in the long term, affecting metabolic, immune and cognitive functions and having an influence on neurological development [19]. *Kenyon *et al*.* found that women who did not take vitamin K had a higher rate of postpartum hemorrhage than women who took vitamin K supplements (45% vs. 12%, respectively) [20]. *Furrer R. *et al*.* found no change in post-partum hemorrhage rate in women with ICP when compared to the control group, leading the authors to conclude that the relevance of vitamin K in ICP post-partum hemorrhage is debatable [21].It should be mentioned that the serum level of vitamin K in enrolled women was not assessed in this study. At the same time, *Maldonado M. *et al. described a severe vitamin K deficiency and ICP-related coagulopathy in a case report, implying a direct relationship between the two [22]. The authors of this case report stated that there was no clear evidence as to whether intestinal malabsorption or insufficient food intake was the cause of hypovitaminosis K. However, the patient's low body mass index led the authors to suspect that a dietary issue was the reason for vitamin K deficiency in this case. On the other hand, *Lees J. *et al*.* did not find cases of coagulopathy in women with ICP; the authors suggest that it a large representative cohort must be studied to determine the true incidence of coagulopathy in ICP [23].

One of the study limitations is the number of participants enrolled in the research. Further large-scale research is needed in order to establish the real incidence of hypovitaminosis K in women with ICP.

## Conclusions

Vitamin K levels have traditionally been assessed indirectly using prothrombin time, prothrombin by Quick and INR values. While a prolonged prothrombin time is a late indicator of vitamin K deficiency, the above markers are more likely to indicate vitamin K activity than its actual level. Vitamin K insufficiency appears to be underreported in the ICP group. To the best of our knowledge, there were no other studies that assessed vitamin K levels in women with ICP. As a result, the authors believe that current study contributes with evidence based scientific knowledge, and provides data regarding vitamin K levels in pregnant women with ICP.

## Data Availability

The datasets used and/or analyzed during the current study available from the corresponding author on reasonable request.

## References

[1] Ozkan S, Ceylan Y, Ozkan OV, Yildirim S. Review of a challenging clinical issue: Intrahepatic cholestasis of pregnancy. World J Gastroenterol 2015; 21(23): 7134–7141 Available from: URL: http://www.wjgnet.com/1007-9327/full/v21/i23/7134.htm10.3748/wjg.v21.i23.7134 10.3748/wjg.v21.i23.7134 PMC4476874 26109799

[2] Williamson C, Geenes V. Intrahepatic cholestasis of pregnancy. ObstetGynecol 2014; 124: 120–133 [PMID: 24901263 10.1097/AOG.0000000000000346] 10.1097/AOG.0000000000000346 24901263

[3] Smith DD, Rood KM (2020). Intrahepatic cholestasis of pregnancy. Clin Obstet Gynecol.

[4] U.S. Department of Health&Human Services, National Institutes of Health, Fact Sheet for Health Professionals: Vitamin K, Updated: June 3, 2020. https://ods.od.nih.gov/factsheets/VitaminK-HealthProfessional/

[5] Booth SL. Vitamin K: food composition and dietary intakes. Food Nutr Res. 2012;56(1):5505. 10.3402/fnr.v56i0.5505. 10.3402/fnr.v56i0.5505 PMC3321250 22489217

[6] Takada T, Yamanashi Y, Konishi K, Yamamoto T, Toyoda Y, Masuo Y, Yamamoto H, Suzuki H (2015). NPC1L1 is a key regulator of intestinal vitamin K absorption and a modulator of warfarin therapy. Sci Transl Med.

[7] Araki S, Shirahata A (2020). Vitamin K deficiency bleeding in infancy. Nutrients.

[8] De Oliveira RB, Stinghen AEM, Massy ZA (2019). Vitamin K role in mineral and bone disorder of chronic kidney disease. Clin Chim Acta.

[9] Ferland G, Erdman JW, Macdonald IA, Zeisel SH (2012). Vitamin K. Present Knowledge in Nutrition.

[10] Zalkhani R, Moazedi A (2020). Basic and clinical role of vitamins in epilepsy. J Res Appliedand Basic Med Sci.

[11] Dowd P, Ham SW, Naganathan S, Hershline R (1995). The Mechanism of Action of Vitamin K. Annu Rev Nutr.

[12] Simes DC, Viegas CS, Araújo N, Marreiros C (2020). Vitamin K as a diet supplement with impact in human health: current evidence in age-related diseases. Nutrients.

[13] Card, D. J., Gorska, R., &Harrington, D. J. (2019). Laboratory assessment of vitamin K status. Journal of Clinical Pathology, jclinpath–2019–205997. 10.1136/jclinpath-2019-205997 10.1136/jclinpath-2019-205997 31862867

[14] Allison AC (2001). The possible role of vitamin K deficiency in the pathogenesis of Alzheimer’s diseaseand in augmenting brain damage associated with cardiovascular disease. Med Hypotheses.

[15] Brown B, Wright C (2020). Safety and efficacy of supplements in pregnancy. Nutr Rev.

[16] Tsuchie H, Miyakoshi N, Hongo M, Kasukawa Y, Ishikawa Y, Shimada Y (2012). Amelioration of pregnancy-associated osteoporosis after treatment with vitamin K2: a report of four patients. Upsala J Med Sci.

[17] Dasari S, Ali SM, Zheng G, Chen A, Dontaraju VS, Bosland MC, Munirathinam G (2017). Vitamin K and its analogs: Potential avenues for prostate cancer management. Oncotarget.

[18] Shahrook S, Ota E, Hanada N, Sawada K, Mori R (2018). Vitamin K supplementation during pregnancy for improving outcomes: a systematic review and meta-analysis. Sci Rep.

[19] Oh C, Keats EC, Bhutta ZA (2020). Vitamin and mineral supplementation during pregnancy on maternal, birth, child health and development outcomes in low-and middle-income countries: a systematic review and meta-analysis. Nutrients.

[20] Kenyon, A. P., Piercy, C. N., Girling, J., Williamson, C., Tribe, R. M., &Shennan, A. H. (2002). Obstetric cholestasis, outcome with active management: a series of 70 cases. BJOG: an international journal of obstetrics and gynaecology, 109(3), 282–288. 10.1016/S1470-0328(02)01368-X 10.1111/j.1471-0528.2002.01368.x 11950183

[21] Furrer, R., Winter, K., Schäffer, L., Zimmermann, R., Burkhardt, T., &Haslinger, C. (2016). Postpartum blood loss in women treated for intrahepatic cholestasis of pregnancy. Obstetrics&Gynecology, 128(5), 1048–1052.10.1097/AOG.0000000000001693 10.1097/AOG.0000000000001693 27741180

[22] Maldonado, M., Alhousseini, A., Awadalla, M., Idler, J., Welch, R., Puder, K., ... &Gonik, B. (2017). Intrahepatic cholestasis of pregnancy leading to severe vitamin K deficiency and coagulopathy. Case Reports in Obstetrics and Gynecology, 2017. 10.1155/2017/5646247 10.1155/2017/5646247 PMC5478816 28680707

[23] Lees J, Al-Rawi S, McPhee H (2019). Coagulopathy in obstetric cholestasis in Wessex Deanery. Int J Obstet Anesth.

